# Conformational Parameters and Hydrodynamic Behavior of Poly(2-Methyl-2-Oxazoline) in a Broad Molar Mass Range

**DOI:** 10.3390/polym15030623

**Published:** 2023-01-25

**Authors:** Alexander S. Gubarev, Alexey A. Lezov, Anna N. Podsevalnikova, Nina G. Mikusheva, Petr A. Fetin, Ivan M. Zorin, Vladimir O. Aseyev, Ondrej Sedlacek, Richard Hoogenboom, Nikolai V. Tsvetkov

**Affiliations:** 1 Department of Molecular Biophysics and Polymer Physics, Saint-Petersburg State University, Universitetskaya Nab. 7/9, 199034 Saint-Petersburg, Russia; 2 Institute of Chemistry, Saint-Petersburg State University, Universitetskaya Nab. 7/9, 199034 Saint-Petersburg, Russia; 3 Department of Chemistry, University of Helsinki, Helsinki, P.O. Box 55, 00014 Helsinki, Finland; 4 Supramolecular Chemistry Group, Centre of Macromolecular Chemistry (CMaC), Department of Organic and Macromolecular Chemistry, Ghent University, Krijgslaan 281 S4, B-9000 Ghent, Belgium

**Keywords:** molecular hydrodynamic, equilibrium rigidity, poly(2-methyl-2-oxazoline), PMeOx, conformation, biomedical applications, thermodynamical solvent quality

## Abstract

In this work, we report our results on the hydrodynamic behavior of poly(2-methyl-2-oxazoline) (PMeOx). PMeOx is gaining significant attention for use as hydrophilic polymer in pharmaceutical carriers as an alternative for the commonly used poly(ethylene glycol) (PEG), for which antibodies are found in a significant fraction of the human population. The main focus of the current study is to determine the hydrodynamic characteristics of PMeOx under physiological conditions, which serves as basis for better understanding of the use of PMeOx in pharmaceutical applications. This goal was achieved by studying PMeOx solutions in phosphate-buffered saline (PBS) as a solvent at 37 °C. This study was performed based on two series of PMeOx samples; one series is synthesized by conventional living cationic ring-opening polymerization, which is limited by the maximum chain length that can be achieved, and a second series is obtained by an alternative synthesis strategy based on acetylation of well-defined linear poly(ethylene imine) (PEI) prepared by controlled side-chain hydrolysis of a defined high molar mass of poly(2-ethyl-2-oxazoline). The combination of these two series of PMeOx allowed the determination of the Kuhn–Mark–Houwink–Sakurada equations in a broad molar mass range. For intrinsic viscosity, sedimentation and diffusion coefficients, the following expressions were obtained: $\left[\eta \right]=0.015M^{0.77}$, $s_{0}=0.019M^{0.42}$ and $D_{0}=2600M^{-0.58}$, respectively. As a result, it can be concluded that the phosphate-buffered saline buffer at 37 °C represents a thermodynamically good solvent for PMeOx, based on the scaling indices of the equations. The conformational parameters for PMeOx chains were also determined, revealing an equilibrium rigidity or Kuhn segment length, (*A*) of 1.7 nm and a polymer chain diameter (*d*) of 0.4 nm. The obtained value for the equilibrium rigidity is very similar to the reported values for other hydrophilic polymers, such as PEG, poly(vinylpyrrolidone) and poly(2-ethyl-2-oxazoline), making PMeOx a relevant alternative to PEG.

## 1. Introduction

Poly(2-oxazoline)s (PAOx) are a class of polymers with tremendous potential for biomedical applications. Even though the conjugation of poly(ethylene glycol) (PEG) to proteins and peptides (PEGylation) remains the most popular technique for blood half-life extension of drugs and carriers, the rather broad occurrence of PEG antibodies in the human population makes it important to develop alternative hydrophilic polymers [1,2,3,4,5].

In recent decades, the main interest in PAOx is related to the fact that this polymer class was shown to be a prospective alternative to PEG for construction of polymer–drug or polymer–protein conjugates [2,6,7,8,9,10,11], while also providing beneficial properties for development of biomaterials and thermoresponsive materials [12,13,14,15,16,17,18].

Studies on the biological and chemical properties of PAOx polymers have demonstrated the biocompatibility, high stability in physiological pH range, chemical and physical versatility, stealth behavior, antifouling characteristics, and good renal clearance of the most common hydrophilic poly(2-oxazoline)s, these being poly(2-methyl-2-oxazoline) (PMeOx) and poly(2-ethyl-2-oxazoline) (PEtOx) [16,19,20,21,22,23].

The variety of 2-oxazoline monomers that are readily available or can easily be synthesized allows access to different polymer architectures and tuning of polymer functionality and properties [24,25,26]. Even though the PAOx appear to be generally biocompatible, this biocompatibility has to be demonstrated for all new derivatives.

To date, PEtOx appears to be the most investigated poly(2-oxazoline) in terms of biomedical applications [7,10,14,15,16,27], and PEtOx based polymer-drug conjugates and hemostatic materials have also reached human clinical trials [28,29,30,31]. Nonetheless, PMeOx is an important runner-up, and is especially attractive to shield drug carriers and for antifouling coatings based on its hydrophilicity, which is higher than that of PEtOx and PEG [17,32,33,34,35,36,37,38,39,40,41,42,43,44]. It was shown that more hydrophilic PMeOx exhibits better anti-fouling properties than both PEtOx with longer side chains and PEG [32,33,34,35,36,37,38], and allows higher hydrophobic drug loading [32].

While comparing particular therapeutically significant properties, thiolated silica nanoparticles functionalized with PMeOx were shown to be considerably more penetrating through mucosal tissue than particles functionalized with PEtOx or poly-(2-n-propyl-2-oxazoline)s [39]. Another recent study showed that modification of bovine pericardium-based bioprosthetic heart valves with PMeOx creates a biocompatible surface that demonstrated enhanced resistance to serum protein infiltration and glycation and better thromboresistance compared to the PEG-modified version [40].

The question regarding the PMeOx and PEtOx polymer chain behavior in solution from a molecular hydrodynamic perspective has previously been addressed by Schubert and Nischang [41]. In order to compare the conformational properties of PMeOx, PEtOx and PEG, the intrinsic viscosities, sedimentation coefficients, frictional ratios and their interrelation were estimated in milliQ water solutions at 20 °C, with subsequent establishment of Kuhn−Mark−Houwink−Sakurada scaling relationships. However, the polymer chain conformation depends on the solvent and its thermodynamic properties, and might be different in pure water, as used in the previous work on PMeOx (limited to a $M_{w}$ of ~20,000 g/mol), and in an aqueous buffer containing salts. In addition, we have previously reported a detailed study on the solution behavior of PEtOx in phosphate buffered saline (PBS) at 37 °C [45].

To the best of our knowledge, the conformation of PMeOx under physiological conditions has not been reported to date. Therefore, the present work is devoted to the comprehensive characterization of the hydrodynamic behavior of PMeOx in PBS as a buffer solution that mimics physiological conditions, since it closely imitates the pH (~7.4), osmolarity, and ion concentrations of the human body. In addition, polymer chain rigidity was determined using a broad range of PMeOx molar masses, up to an $M_{w}$ of ~70,000 g/mol both in water at 20 °C and in PBS solution at 37 °C.

PAOx are usually prepared by living cationic ring-opening polymerization (LCROP) of 2-oxazolines, which allows the preparation of well-defined polymers with narrow molar mass distribution [24,46]. However, attempts to prepare defined high molar mass PMeOx via LCROP of its monomer failed due to extensive chain transfer and chain-coupling side reactions [47]. Therefore, some of us have recently proposed a novel alternative strategy for the preparation of high-molar-mass PMeOx based on acetylation of well-defined linear polyethyleneimine (PEI) prepared by controlled side-chain hydrolysis of defined high molar mass PEtOx [47]. This strategy allowed us to prepare low-dispersity PMeOx with a molar mass of up to ~70,000 g/mol, reaching the range of molar masses suitable for polymer–drug conjugates’ construction which is limited by the renal excretion threshold that has been determined to be around 40,000–50,000 g/mol for PEtOx and PEG, and will most likely be in a similar range for PMeOx [22]. Considering molecular hydrodynamics, this broader molar mass range is essential for scaling relationships, and has allowed accurate determination of the conformational parameters of PMeOx for the first time.

The following article is organized as follows. First, the determination of the molecular hydrodynamic parameters of the PMeOx samples will be discussed; second, their intercorrelation and consistency is checked with both the hydrodynamic invariant concept and the interrelation of Kuhn–Mark–Houwink–Sakurada scaling indices; third, the most comprehensive hydrodynamic models are applied for estimation of conformational parameters of the PMeOx chains; fourth, the hydrodynamic behavior of PMeOx will be discussed in relation to the pharmaceutical closest polymer alternatives, namely PEG, PEtOx and poly(vinylpyrrolidone) (PVP).

## 2. Materials and Methods

### 2.1. Materials

**Figure 1:**
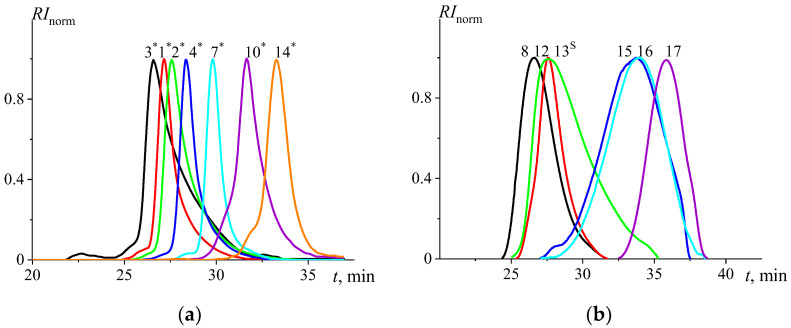
The distributions are obtained with SEC: (**a**)—the PMeOx samples are eluted with DMA/LiCl and (**b**)—DMF/LiBr. The numbers next to the distributions correspond to the sample numbering.

For this study, two sets of PMeOx were investigated. The first set was prepared by LCROP using an optimized protocol at low temperature in chlorobenzene to suppress chain-transfer side reactions [46,48]. As the direct LCROP of MeOx does not allow the preparation of defined high molar mass PMeOx, a second set of PMeOx was prepared by acetylation of well-defined linear poly(ethylene imine) prepared by controlled side-chain hydrolysis of defined high molar mass PEtOx [47]. These PMeOx obtained by acetylation will be indicated with an asterisk in this work, e.g., PMeOx*. ^1^H-NMR spectroscopy confirmed the PMeOx structure and the first estimations of molar masses for these synthesized PMeOx were made by SEC-MALS (Figure 1 and Table 1). In addition, two commercially available hydroxy-terminated PMeOx samples were studied; the first was purchased from Sigma-Aldrich (Burlington, USA, cat. # 795283) and the second kindly provided by Ultroxa (Ghent, Belgium, prod. code: HR11.0100/0101). These are marked with upper index ‘S’ and ‘U’, correspondingly.

It should be noted that all of the studied PMeOx samples were found to be hygroscopic. Therefore, all the samples were dried under vacuum (~30 mbar) at 55 °C up to constant weight value of a sample (~3 h) before standard solution preparation procedure. The sample weighing revealed up to 13% difference in the sample weight before and after vacuum drying, corresponding to the loss of water. The solution concentration is proportional to the weight of a sample, so it must be determined correctly based on unperturbed sample weight.

The investigations of the molecular hydrodynamic methods (intrinsic viscosity, velocity sedimentation and dynamic light scattering) were carried out in a PBS buffer at 37 °C and milliQ water at 20 °C. It was prepared with ultrapure water obtained with Millipore (Direct-Q^®^ 8 UV) and standard phosphate-buffered saline tablets (1 tablet/200 mL) purchased from Sigma-Aldrich. The initial water was characterized with a resistivity value of 18.2 MΩ cm and $\mathrm{pH}=\left(7.0\pm 0.1\right)$, which was determined at 25.0 °C with laboratory ionomer/conductometer/oxygenometer Anion-4151. The prepared PBS solution showed $\mathrm{pH}=\left(7.57\pm 0.01\right),$ and no further pH value adjustments were done. The densities and dynamic viscosities of PBS were experimentally determined and their values constituted as follows: $\rho_{0}\left(37\;{}^{\circ}C\right)=1.00012\;g/{\mathrm{cm}}^{3}$ and $\eta_{0}\left(37\;{}^{\circ}C\right)=0.709\;\mathrm{cP}$, correspondingly. The following solvent parameters were used for treating the experimental data at H_2_O 20 °C: $\rho_{0}\left(20\;{}^{\circ}C\right)=0.9982\;g/{\mathrm{cm}}^{3}$ and $\eta_{0}\left(20\;{}^{\circ}C\right)=1.002\;\mathrm{cP}$.

### 2.2. Methods

*Size exclusion chromatography (SEC)*: SEC was used for initial estimations of the molar masses ($M_{w}$—weight-averaged and $M_{n}$—number-averaged) together with the dispersity values (*Đ* $=M_{w}/M_{n}$) of the prepared polymers. This was performed using an Agilent 1260-series (Agilent Technologies Inc., Santa Clara, CA, USA) high-performance liquid chromatography (HPLC) system equipped with a 1260 ISO-pump and a 1260 automatic liquid sampler. The column compartment was thermostated at 50 °C and equipped with two PLgel 5 μm mixed-D columns and the precolumn (PLgel 5 µm Guard, 50 × 7.5 mm) in series. The 1260 diode array detector, 1260 RI detector and multi-angle light scattering detector (Wyatt miniDawn Treos II) were engaged in data collection. The used eluent was *N*,*N*-dimethylacetamide (DMA) containing 50 mM of LiCl. The flow rate was 0.5 mL/min. The molar mass values and *Đ* values were calculated using refractive index increments dn/dc obtained with a differential refractometer (Wyatt Optilab T-rEX) (Table 1 and Figure 1a).

The other series of samples was studied on a Shimadzu LC-20AD (Shimadzu Corp., Kyoto, Japan) chromatograph equipped with a refractive index detector and TSKgel Guard, G5000HHR, and G2500HHR columns (Tosoh Bioscience, Tokyo, Japan). The analysis conditions were set accordingly: 0.1 M LiBr in dimethylformamide (DMF), 60 °C, 0.5 mL/min. Then, the polymer solution in 0.1 M LiBr DMF (~6 mg/mL) was eluted through the experimental setup. Calculation of the average molecular weight and the polydispersity index was carried out according to the cubic calibration dependence in the Shimadzu LCsolution program, using polystyrene standards: 500–238,000 g/mol (Table 1 and Figure 1b).

*Viscometry measurements:* Intrinsic viscosities $\left[\eta \right]$ were determined with the data obtained using a Lovis 2000 M microviscometer (Anton Paar GmbH, Graz, Austria). The experiments were based on the rolling ball (Höppler) principle; standard dilution procedures were used. The setup included a capillary with an inner diameter of 1.59 mm and equipped with a gold-coated steel ball (1.50 mm in diameter). The rolling times for a solvent ($t_{0}$) and polymer solutions of various concentrations ($t_{c}$) were measured at a tilting angle of the capillary of 45°, within the wide range of solution concentrations c and a temperature range of 20≤T,°C≤60.

*Densitometry:* Density measurements were carried out in the pure water at T=37 °C using the density meter DMA 5000 M (Anton Paar GmbH, Graz, Austria) and according to the procedure described in Kratky et al. [49]

*Analytical ultracentrifugation (AUC):* Velocity sedimentation experiments were performed with a ProteomeLab XLI Protein Characterization System analytical ultracentrifuge (Beckman Coulter, Brea, CA, USA) using conventional double-sector or aluminum centerpieces with 12 mm optical path length and a four-hole analytical rotor (An-60Ti). The rotor speeds were 50,000–55,000 rpm depending on the sample. Cells were filled with 420 μL of a sample solution and 440 μL of the PBS buffer. Before the run, the rotor with installed centerpieces was thermostated for approximately 2 h at 37 °C in the centrifuge-vacuumed chamber. Sedimentation profiles were obtained at the same temperature using interference optics.

For the analysis of the velocity sedimentation data, the Sedfit program was used [50]. The continuous $c\left(s\right)$ distribution model implemented within Sedfit coupled with a Tikhonov–Phillips regularization procedure allows us to obtain the differential distribution on sedimentation coefficients s and the frictional ratio $\left(f/f_{\mathrm{sph}}\right)$ value, thus determining the averaged diffusion coefficient of sedimenting species. The $c\left(s\right)$ analysis is based on numerical solution of the Lamm equation, assuming the averaging frictional ratio values for all the species involved in the process. The Lamm equation is a partial differential equation [51]:

(1) $$\frac{dc}{dt}=\frac{1}{r}\frac{d}{dr}\left(rD\frac{dc}{dr}-s\omega^{2}r^{2}c\right),$$

where t is the time of applying the centrifugal field at the distance r with a rotor rotating at an angular speed ω. The first term of the equation describes the diffusion process at the created solution–solvent boundary, which is formed due to the centrifugal field and sedimenting species (second term); then, D and s are the diffusion and sedimentation coefficients, correspondingly. Thus, the Sedfit numerically solves Equation (1) within the given parameters and searches for the least-residual values between the experimental data and resolved solution.

*Dynamic Light Scattering (DLS):* The DLS study of the series of PMeOx homologous in PBS at 37 °C was carried out using a “PhotoCor Complex” spectrometer (Photocor Instruments Inc., Moscow, Russia). The apparatus is based on a digital correlator (288 channels, 10 ns), a standard goniometer (10°–150°), and a thermostat with temperature stabilization of 0.05 °C. A single-mode linear polarized laser (wave length $\lambda_{0}=405$ nm) was used as an excitation source; the experiments were carried out at scattering angles (ϑ) ranging from 30° to 130°. Autocorrelation functions of scattered light intensity were processed using the inverse Laplace transform regularization procedure incorporated in DynaLS software (provided by Photocor Instruments Inc., Moscow, Russia), which provides distributions of scattered light intensities by relaxation times $\rho \left(\tau \right)$. The dependence of 1/τ (where τ is the position of a maximum of the $\rho \left(\tau \right)$ distribution) on the scattering vector squared was calculated as $q^{2}={\left(\left(4\pi n/\lambda_{0}\right)\sin\left(\vartheta /2\right)\right)}^{2}$; here, n is the refractive index of a solvent. For all studied samples, this was a straight line passing through the origin, indicating the translation diffusional character of the observed processes ($1/\tau =Dq^{2}$) [52,53,54]. Diffusion coefficients D, obtained at finite concentration c, were extrapolated to an infinite dilution limit to determine their unperturbed value $D_{0}$. This was accomplished according to the equation $D=D_{0}\left(1+2A_{2}Mc+\ldots \right)$, where $A_{2}$ is the second virial coefficient. The hydrodynamic radius $R_{h}$ was calculated using the Stokes–Einstein equation [55]:

(2) $$R_{h}=\frac{k_{B}T}{6\pi \eta_{0}D_{0}},$$

where $k_{B}$ is the Boltzmann constant and T is the absolute temperature on the Kelvin scale.

## 3. Results

SEC analysis was performed for initial evaluation of the molar mass range and dispersity values of the PMeOx samples obtained by both LCROP and acetylation of PEI. The results are summarized in Table 1 and SEC traces are shown in Figure 1, indicating the investigated PMeOx cover a wide range of molar masses from $1.8<M_{w}10^{3},\;g/\mathrm{mol}<70,$ with *Ɖ* values around 1.1–1.4. These values should be treated as initial estimates; true values are obtained with sedimentation-diffusion analysis further in the study, which represent the absolute molar mass values. A similar discrepancy within $M_{w}$ obtained with SEC and absolute molar masses was also reported earlier [41,56].

**Figure 2:**
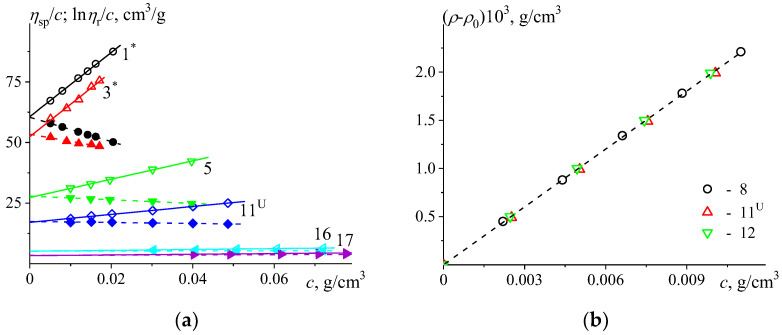
(**a**): The normalized specific viscosity $\eta_{sp}/c$ (open symbols) and natural logarithm of relative viscosity $\ln\eta_{r}/c$ (filled symbols) vs. concentration c in PBS at 37 °C; (**b**): the dependence of $\left(\rho -\rho_{0}\right)$ vs. concentration c in H_2_O at 37 °C, obtained for the studied PMeOx samples. The numbers next to the data points correspond to sample numbering.

The data obtained in the study of viscous flow of the PMeOx solutions were treated within the frameworks of Huggins and Kraemer equations [57,58]. For accomplishing this goal, the dependences of specific viscosity $\eta_{\mathrm{sp}}=\left(t_{c}/t_{0}-1\right)$ and natural logarithm of relative viscosity $\ln\eta_{r}=\ln\left(t_{c}/t_{0}\right)$ were normalized by the solution concentrations c (Figure 2a and Figure S1). It should be mentioned that in a general case, $\eta_{\mathrm{sp}}=\left(\eta_{c}/\eta_{0}-1\right)$ and $\ln\eta_{r}=\ln\left(\eta_{c}/\eta_{0}\right)$ (where $\eta_{c}$ is the dynamic viscosity of a solution with a concentration c); however, according to the conditions of the performed experiments, $t_{c}/t_{0}=\eta_{c}/\eta_{0}$. Then, the concentration dependences of $\eta_{\mathrm{sp}}/c\left(c\right)$ as well as $\ln\eta_{r}/c\left(c\right)$ were extrapolated to zero concentration resulting in intrinsic viscosity values $\left[\eta \right]$ together with Huggins k′ and Kraemer k″ parameters (Table 2). The intercept values with the Y-axis correspond to intrinsic viscosity $\left[\eta \right]$ values, and the the slope values of the corresponding linear dependences allow the calculation of Huggins and Kraemer parameters.

The dependences ($\eta_{\mathrm{sp}}/c\left(c\right)$ and $\ln\eta_{r}/c\left(c\right)$) were obtained in the region of diluted polymer solutions ($t_{c}/t_{0}<2.5$) and demonstrated linear behavior in the studied concentration range. The intrinsic viscosities obtained with Huggins ${\left[\eta \right]}_{H}$ and Kraemer ${\left[\eta \right]}_{K}$ equations were found to be in good agreement with each other within the experimental error. Therefore, the averaged values were used in the further analysis. The values of intrinsic viscosity determined for the PMeOx samples are typical of moderate molar mass linear polymers. The averaged Huggins parameter $k^{\prime }$ equals (0.46 ± 0.02), suggesting that the studied system herein is still in thermodynamically good conditions, with a tendency to worsen (the $k^{\prime }$, $k^{\prime \prime }$ values determined for the lowest molar mass samples were not included in averaging) [57,59]. It can be noted that with decreasing polymer chain length, the Huggins parameter increases (Table 2). The known interrelation of Huggins and Kraemer parameters $k^{\prime }-k^{\prime \prime }=0.5$ follows the purely mathematical dependences of Huggins and Kraemer equations’ expansion in the series. In our case, it is not fully satisfied, and the averaged value of Kraemer parameter $-k^{\prime \prime }=\left(0.11\pm 0.01\right)$ leads to $k^{\prime }-k^{\prime \prime }=\left(0.57\pm 0.02\right)$. Whenever it was not possible to proceed with study of concentration dependence, the first approximation the $\left[\eta \right]$ value was estimated by one concentration using the Solomon-Cuita equation [60].

Additionally, the temperature dependence of PMeOx viscosity in pure H_2_O has been studied at the following T values: 20, 37 and 60 °C (Tables S1 and S2 and Figure S1). The trend towards decreasing of intrinsic viscosity is observed, although it is much less pronounced than that determined earlier for PEtOx macromolecules [45]. It is in a good agreement with the fact that lower critical solution temperature (LCST) behavior was determined for the closest relative structures of PMeOx, such as PEtOx, poly(2-n-propyl-2-oxazoline) and poly(2-isopropyl-2-oxazoline) [61,62]. The PMeOx studied herein did not show any change in transmittance within the studied molar mass, concentration and temperature range.

Comparison of intrinsic viscosity values of the same PMeOx samples obtained in H_2_O and PBS at the same temperature T=37 °C shows that they are very close and found within doubled experimental error values (Table S2). Yet, all the $\left[\eta \right]$ values characterizing PMeOx macromolecules at PBS medium demonstrate lower values, which may indicate the specific influence of bulky PBS ions over polymer coils.

Density measurements are necessary for quantitative interpretation of analytical ultracentrifugation data. The density increment $d\rho /dc=\left(1-\;\bar{\upsilon }\rho_{0}\right)$ ($\bar{\upsilon }$ is partial specific volume of the polymer, $\rho_{0}$ is solvent density) was determined by consistent dilution of the PMeOx solutions, leading to the average partial specific volume $\bar{\upsilon }=\left(0.804\pm 0.002\right)$ cm^3^/g over all studied samples (Figure 2b). The obtained value was found to be in good agreement with the previously independently obtained value $\bar{\upsilon }=\left(0.81\pm 0.01\right)$ cm^3^/g for same system, PMeOx-H_2_O at 20 °C [41]. It should be noted that both the viscometry and densitometry measurements are highly sensitive to the effects of hygroscopy. Thus, previously described measures must be taken to ensure that the sample concentration is determined reliably; for this, we ensured extensive drying of the PMeOx samples before analysis.

**Figure 3:**
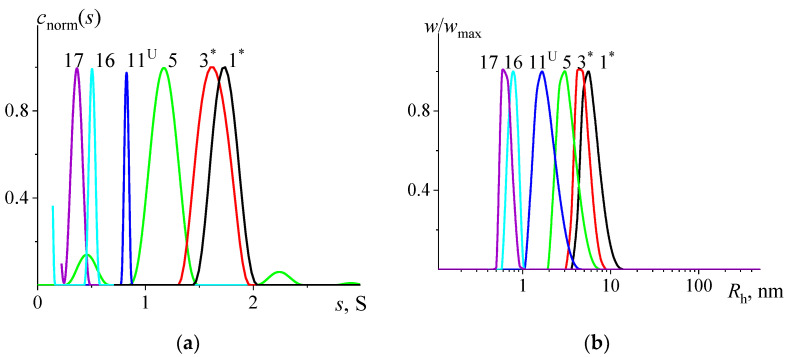
(**a**): The normalized $c_{\mathrm{norm}}\left(s\right)$ distributions vs. sedimentation coefficients s resolved with Sedfit at the lowest studied concentrations c≈0.025 g/dL and (**b**): the normalized distributions of weight–component concentration $w/w_{max}$ over hydrodynamic radii $R_{h}$. Both $c_{\mathrm{norm}}\left(s\right)$ and $w/w_{max}$ are obtained for PMeOx solutions in PBS at 37 °C. The numbers next to the distributions correspond to the sample numbering.

The velocity sedimentation method accomplished upon analytical ultracentrifuge allows the resolution of acquired data using continuous $c\left(s\right)$ distribution with the Sedfit program (Figure 3a). The majority of obtained distributions demonstrated a mono peak, and fewer showed multi-modal distribution, which is a Sedfit artifact related to the finite dispersity value *Đ* of studied samples. Practically all the distributions demonstrated a low s value peak/shoulder next to Y-axis at s≲0.2 S, which might appear while studying low molar mass samples or can be an indication of the presence of impurities. Such peaks were ignored in further analysis.

As a result, the set of sedimentation coefficients and frictional ratio values were obtained at the studied PMeOx solution concentrations. Regularly both of the characteristics are concentration dependent and require extrapolation to zero concentration, where sedimentation coefficient $s_{0}$ and frictional ratio $\left(f/f_{\mathrm{sph}}\right){\;}_{0}$ values within the infinite dilution limit can be determined (Figure S2a,b). The extrapolations were made with following equations:

(3) $$s^{-1}=s_{0}^{-1}\left(1+k_{s}c+\ldots \right),$$

(4) $$(f/f_{sph})={\left(f/f_{sph}\right)}_{0}\left(1+k_{f}c+\ldots \right),$$

where $k_{s}$ is the Gralen coefficient and $k_{f}$ is the concentration frictional ratio parameter. At least three concentrations of each sample were studied, covering a wide concentration range $c_{\max}/c_{\min}>3$). The dimensionless parameter $c\left[\eta \right],$ characterizing the degree of dilution, was in the range of $0.1\;\leq \;c\left[\eta \right]10^{2}\;\leq \;5.0$, corresponding to a high dilution state; this, in turn, allows reliable determination of unperturbed $s_{0}$ and $\left(f/f_{\mathrm{sph}}\right){\;}_{0}$ values in the infinite dilution limit. The corresponding data were well fitted with linear dependences. For the majority of PMeOx low molar mass samples (PMeOx 5–17), the determined $s_{0}$ did not demonstrate concentration dependence within a studied concentration range and its value had negligible deviations from its average value within an experimental error.

DLS results are presented in Figure S3a, which shows normalized scattered light intensity distributions on hydrodynamic radii. Before the study, the solutions were centrifuged at 15,000 rpm for 15 min to eliminate the effect of large impurities. For most samples, the $I\left(R_{h}\right)$ distributions were unimodal, but for low molar mass samples (PMeOx-15 to 17), the presence of particles with a hydrodynamic radius of 10–100 nm was observed (Figure S3a). The concentration of these particles in the solution was estimated from the assumption that the scattered light intensity is proportional to the product of the mass of the particles and its concentration I~cM, and the mass is proportional to the radius to the power of α. The exponent α depends on the shape of the scattered particles. Assuming that the shape of the studied particles is close to spherical, the value α=3 was used. Such estimations show that the concentration of large particles in the solutions of the studied systems did not exceed one percent (Figure 3b). Thus, it can be concluded that the large components of the solution should not distort the results obtained either by the DLS method or other methods used in the paper. The concentration dependences are presented in Figure S3b.

The diffusion coefficient values D were obtained directly with DLS measurements and by calculations with frictional ratio values $\left(f/f_{\mathrm{sph}}\right){\;}_{0}$ evaluated with AUC $c\left(s\right)$ analysis:

(5) $$D_{0\mathrm{sf}}=\frac{k_{B}T}{9\pi \sqrt{2}}{\left(\frac{1-\bar{\upsilon }\rho_{0}}{\eta_{0}^{3}{\left(f/f_{sph}\right)}_{0}^{3}s_{0}\bar{\upsilon }}\right)}^{1/2}.$$

The $D_{0\mathrm{sf}}$ values obtained with Equation (5) should be treated very carefully, as these values are the fitted parameter of direct experimentation on the determination of sedimentation coefficient. However, as was also demonstrated earlier, if the obtained $D_{0\mathrm{sf}}$ values are found reasonable within the concept of the hydrodynamic invariant, then both diffusion coefficients obtained with DLS and calculated with AUC data can be averaged to increase the accuracy of its determination [41,45,63,64].

This analysis can be performed by fixing intrinsic viscosity with sedimentation coefficient values and varying diffusion coefficient through a well-known expression for hydrodynamic invariant $A_{0}$ [65]:

(6) $$A_{0}={\left(R\left[s\right]{\left[D\right]}^{2}\left[\eta \right]\right)}^{\frac{1}{3}},$$

where R is the universal gas constant, $\left[s\right]\equiv s_{0}\eta_{0}/\left(1-\bar{\upsilon }\rho_{0}\right)$ and $\left[D\right]\equiv D_{0}\eta_{0}/T$ are the characteristic sedimentation and diffusion coefficients, correspondingly, which are independent from the common solvent properties of dynamic viscosity and density. The results are presented in Table S3. The calculations result in the following values $A_{0}=\left(3.2\pm 0.3\right)$ and $A_{0\mathrm{sf}}=\left(3.7\pm 0.4\right)$ $g\;{\mathrm{cm}}^{2}/\left(s^{2}{K\;\mathrm{mol}}^{1/3}\right)$ averaged over all studied PMeOx samples. It can be seen that the obtained values are well correlated and appear indistinguishable within the experimental error. This means that the diffusion coefficients obtained with independent techniques can be averaged, and the average values were used in further analysis. The average value of the hydrodynamic invariant obtained with averaged diffusion coefficient values results in $A_{0}=\left(3.5\pm 0.3\right)$ $g\;{\mathrm{cm}}^{2}/\left(s^{2}{K\;\mathrm{mol}}^{1/3}\right)$. The calculated value is typical of experimental values of hydrodynamic invariants known for flexible chain polymers in thermodynamically good solvents.

Table 3 contains the main hydrodynamic parameters, hydrodynamic invariants estimated with Equation (6) and molar masses determined with the Svedberg equation:

(7) $$M_{sD}=\frac{s_{0}RT}{D_{0}\left(1-\bar{\upsilon }\rho_{0}\right)}=\frac{\left[s\right]}{\left[D\right]}R.$$

Now, as the initial set of hydrodynamic parameters is obtained, the consistency of the hydrodynamic data is established and the molar masses are determined, it is possible to move on to the Discussion section.

## 4. Discussion

### 4.1. Kuhn–Mark–Houwink–Sakurada Equations

**Figure 4:**
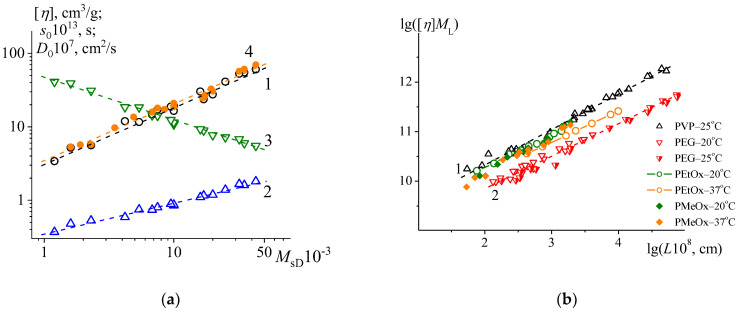
(**a**): The double logarithmic plot of KMHS relationships, for intrinsic viscosity (1), sedimentation coefficients (2) and diffusion coefficients (3) obtained for PMeOx samples in PBS solutions at 37 °C, (4) represents the combined analysis of the viscometry data obtained herein and in earlier studies (ref. [41]) for PMeOx in H_2_O solutions at 20 °C. The equation parameters are presented in Table 4. (**b**): The specific hydrodynamic volume plot, representing data for relevant water soluble polymers: PVP in 0.1 M sodium acetate at 25 °C [66]; PEG disregarding end-groups in H_2_O at 20 °C [67] and 25 °C [68,69]; PEtOX in H_2_O at 20 °C [41] and PBS at 37 °C [45]; PMeOx in H_2_O at 20 °C, is combined data of the research herein [41], and PMeOx in PBS at 37 °C is from the data of the current study; lines (1) and (2) are the linear extrapolation of PVP and PEG data.

The hydrodynamic parameters describing a polymer homologous series in certain conditions (solvent, temperature, the ion strength of a solution, etc.) are interrelated with each other, and molar mass is found through the canonical Kuhn–Mark–Houwink–Sakurada (KMHS) equation, which can be presented in the following form:

(8) $$P_{i}=K_{\mathrm{ij}}P_{j}^{b_{\mathrm{ij}}},$$

where $P_{i}$ is one of the hydrodynamic characteristics $\left[\eta \right]$, $D_{0}$, $s_{0}$ and $P_{j}$ is another corresponding hydrodynamic characteristic or corresponding molar mass. The results of treating three pairs of data sets ($\left[\eta \right]-M_{\mathrm{sD}}$, $s_{0}-M_{\mathrm{sD}}$ and $D_{0}-M_{\mathrm{sD}}$) are presented in Figure 4a, and corresponding scaling indices $b_{\mathrm{ij}}$ and coefficients $K_{\mathrm{ij}}$ are collected in Table 4.

The scaling indices of the KMHS equation are interrelated with each other through the following expressions: $\left|b_{D}\right|=\left(1+b_{\eta }\right)/3$ and $\left|b_{D}\right|+b_{s}=1$, so if the expressions are satisfied, the consistency of the obtained indices is established. Through the first expression, the absolute value of $b_{D}$ is calculated; it equals (0.59±0.01), which correlates well with the extrapolated value $\left|b_{D}\right|=\left(0.58\pm 0.02\right)$. The second expression is also satisfied. The obtained KMHS equation parameters are in satisfactory agreement with the results of the previous PMeOx study [41] taking into account different molar mass range and used solvent. In fact, the combined analysis of the viscometry data obtained herein and previously [41] in the same conditions (H_2_O at 20 °C) allows us to specify the KMHS equation coefficient and scaling indices (Table 4). Thus, the consistency of the KMHS equations is ensured, and it is possible to proceed to the analysis of the conformational characteristics of PMeOx.

### 4.2. Conformation Analysis

The scaling indices of the KMHS equations obtained for the PMeOx homologous series in PBS at 37 °C are higher than the characteristic values for θ-conditions ($b_{\eta }=b_{s}=\left|b_{D}\right|=0.5$). This relatively high value of the exponent may be due to excluded volume effects, as well as the polymer draining effect. Thus, the analysis of the homologous series of these polymers should be carried out taking into account these two contributions. There is a set of hydrodynamic theories that makes it possible to calculate the conformational characteristics of polymers, taking into account excluded volume interactions and draining effects to varying degrees. This can be done by analyzing the data of rotational friction (viscometry) and translational friction (velocity sedimentation and diffusion) processes, which allow us to determine the corresponding parameters of equilibrium rigidity $A_{\eta }$, $A_{D}$ and the effective hydrodynamic diameter $d_{\eta }$, $d_{D}$ of a polymer chain depending on the extrapolated type of data (indices η—rotational friction data and D—translational friction data). To acquire the conformation parameters, the molar mass per unit chain length $M_{L}=M_{0}/\lambda$ must be determined. Here, $M_{0}$ is the molar mass of a monomer unit of PMeOx, and λ is the projection of a monomer unit in the direction of a fully extended polymer chain. $M_{0}$ is calculated based on the polymer unit structure and equals 85.11 g/mol, and $\lambda =3.78\times 10^{-8}\;\mathrm{cm}$ is the known value characterizing the alkyl chain. This leads to the result of the molar mass per unit chain length calculation, $M_{L}=2.25\times 10^{9}$ g/(mol cm). Additionally, the parameter characterizing thermodynamic quality of the solvent ε must be determined. This can be done by assessing the scaling indices of the KMHS equations $\varepsilon =\left(2b_{\eta }-1\right)/3=\left(2\left|b_{D}\right|-1\right)$, and results in an average value $\varepsilon =\left(0.17\pm 0.03\right)$ for PBS at 37 °C.

The Yamakawa–Fujii theory [70] allows us to calculate the equilibrium rigidity and effective hydrodynamic diameter of a polymer chain based on the model of a worm-like spherocylinder. Within the framework of this approach, the macromolecule is represented as a Kratky–Porod persistence chain [55]; the contribution of excluded volume effects is not taken into account in this model (Figure S4 and Table 5).

The other limiting cases are the approaches described by Fixman–Stockmayer [71] for the rotational friction process and Cowie–Bywater [72] for the translational friction process (Figure S5 and Table 5). They are based on the fact that the contribution of excluded volume interactions to the conformation of a polymer macromolecule increases simultaneously with its molar mass. The procedure for determining the equilibrium characteristics, therefore, involves an extrapolation of the experimentally determined hydrodynamic characteristics of the macromolecule to the limit of low molar mass, where volume effects are negligible.

Within the most sophisticated Gray–Bloomfield–Hearst theory [73], both contributions are taken into account, which makes it possible to calculate the main conformational parameters of the polymer, i.e., the equilibrium rigidity and the effective hydrodynamic diameter of the polymer chain (Figure S6 and Table 5). In addition, this can be done by assessing both translational friction processes data within the framework of initial theory and rotational friction data using the substitute suggested in [66]. Such calculations enable the comparison of the equilibrium properties of the studied series of polymers, with their chemical and molecular-hydrodynamic analogues.

The first and second approaches described above are not fully universal. The first case is characterized by an overestimation of the equilibrium rigidity, since volume effects are not taken into account. The second approach is valid only for sufficiently long macromolecules. However, a comparison of the results of all three methods for calculating the conformational characteristics of polymers allows us to conclude the nature of the role of excluded volume and draining effects in the studied polymer system. Thus, the first and second approaches allow us to assess the range of expected equilibrium rigidity of 1.3<A, nm<2.7. On the other hand, the Gray–Bloomfield–Hearst theory allows us to determine equilibrium rigidity more accurately, leading to $A=\left(1.7\pm 0.2\right)\;\mathrm{nm}.$ The same procedure of conformational parameters’ acquisition was performed with the independently obtained data on PMeOx-H_2_O (20 °C) in the previous reported study [41], which essentially results in a practically indistinguishable equilibrium rigidity value of $A=\left(1.7\pm 0.2\right)\;\mathrm{nm}$ (Table S4). The determined value correlates well with other relevant water soluble polymers, poly(ethylene glycol) $A_{\mathrm{PEG}}=\left(1.9\pm 0.2\right)\;\mathrm{nm}$ [68], poly(vinylpyrrolidone) (PVP) $A_{\mathrm{PVP}}=\left(2.0\pm 0.4\right)\;\mathrm{nm}$ [66] and poly(2-ethyl-2-oxazoline) (PEtOx) $A_{\mathrm{PEtOx}}=\left(1.8\pm 0.3\right)\;\mathrm{nm}$ [45]. This means that analogous hydrodynamic behavior can be expected for the above listed polymer systems within the studied temperature range. The obtained value $A_{\mathrm{PMeOx}}=1.7\;\mathrm{nm}$ allows us to estimate the number of segments entering the PMeOx macromolecules within a studied molar range, as $N=L/A=M/\left(M_{L}A\right)$ (Table 3). The obtained values mean that the study is performed within a large range of specific chain lengths from a few segments to the fully formed polymer coil.

Another conformational parameter, *viz.* the diameter of a polymer chain, is determined with large experimental error within the following range: 0.2<d,nm<0.4. The accuracy of determination of this value within the frameworks of applied theories (Table 5) can be checked through the following equation, interrelating the partial specific volume with d through a purely geometrical assumption by representing the molar mass per unit of chain length as a cylinder with uniformly distributed material [74]:

(9) $$d=\sqrt{\frac{4M_{0}\bar{\upsilon }}{\pi \lambda N_{A}}},$$

where $N_{A}$ represents Avogadro’s constant. Equation (9) leads to $d=\left(0.62\pm 0.01\right)\;\mathrm{nm}$. This value correlates well within the upper limit of the obtained experimental error of d values (Table 5). So, the average d value, based on all assessed values, can be calculated to equal (0.4 ± 0.2) nm.

### 4.3. The Specific Hydrodynamic Volume of Relevant Water Soluble Polymers

The visual representation of equivalency in hydrodynamic behavior of relevant water-soluble polymers (PEG, PVP, PEtOx and PMeOx) can be ensured through the concept of the specific hydrodynamic volume [75,76]. However, to accomplish this goal while comparing the different polymer structures, the fundamental Flory–Fox equation $\left[\eta \right]=\Phi_{0}\left({\langle h^{2}\rangle }^{3/2}/M\right)$ has to be transformed, to account for the structural parameter *viz.,* the mass per unit length $M_{L}$. Thus, multiplying the both parts of the equation results in the following coordinates: $\left[\eta \right]M_{L}=\Phi_{0}\left({\langle h^{2}\rangle }^{3/2}/L\right)$, where the product of $\left[\eta \right]M_{L}$ is proportional to the hydrodynamic volume ${\langle h^{2}\rangle }^{3/2}$. The results are presented in Figure 4b, where the polymer systems in consideration are distributed within the area limited by line (1) showing the extrapolation of PEG data, taking the lowest hydrodynamic volume, and line (2) describing PVP data and showing the upper limit of the hydrodynamic volume values of the analyzed structures. The decrease in the hydrodynamic volume of PEtOx in PBS at 37 °C is most probably associated with the demonstration of worsening thermodynamic quality in the solutions.

The polymer systems in considered coordinates are mainly distributed according to the macromolecular size in solution, which primarily depends on the equilibrium rigidity of the polymer chains and thermodynamic quality of the solutions. The thermodynamic qualities of the solutions are represented by the slopes of the dependences, which are determined by $b_{\eta }$ values in these coordinates. The equilibrium rigidities of the structures were discussed above, and there were no distinct differences determined. Thus, the specific hydrodynamic volumes of the analyzed structures may be considered virtually identical. The lower values of hydrodynamic volume characterizing PEG might be explained by the absence of side groups.

## 5. Conclusions

All of the current study goals described in the introduction have been addressed. The two series of PMeOx samples, synthesized by conventional living cationic ring-opening polymerization and strategy based on acetylation of well-defined linear PEI prepared by controlled side-chain hydrolysis of defined high molar mass of poly(2-ethyl-2-oxazoline), were characterized using molecular hydrodynamic methods including viscometry, analytical ultracentrifugation and dynamic light scattering. The self-consistency of the initially acquired hydrodynamic characteristics was checked within the concept of the hydrodynamic invariant and resulted in $A_{0}=\left(3.5\pm 0.3\right)$ $g\;{\mathrm{cm}}^{2}/\left(s^{2}{K\;\mathrm{mol}}^{1/3}\right)$. The calculated value is typical of experimental values of hydrodynamic invariants known for flexible chain polymers in thermodynamically good solvents. The scaling indices of the obtained KMHS have also confirmed the status of a thermodynamically good solvent quality for the studied system. The interrelation of scaling indices of the KMHS equations was also found to be satisfactory. The conformational parameters for PMeOx chains have been discussed in detail, with implementation of all modern theories describing the processes of rotational and translational friction accomplished in molecular hydrodynamic experiments. The obtained values of equilibrium rigidity and Kuhn segment length of A=1.7 nm and diameter d=0.4 nm characterize PMeOx as a flexible chain polymer with hydrodynamic properties recognized in contiguous water-soluble polymers such as PEG, PVP and PEtOx. 

## 6. Patents

R.H. is listed as inventor on the patent application WO2016008817A1 that covers the investigated defined high molar mass polymers.

## Figures and Tables

**Table 1:** The table contains SEC-MALS data on weight-average $M_{w}$, number-average $M_{n}$ and polydispersity *Đ* values of the studied poly(2-methyl-2-oxazoline) (PMeOx) samples. The last column contains true molar masses $M_{\mathrm{sD}}$ obtained further in the study by sedimentation-diffusion analysis, which represents the absolute technique for determination of molar masses.

PMeOx Sample	$M_{w}10^{-3}\;[g/\mathrm{mol}]$	$M_{n}10^{-3}\;[g/\mathrm{mol}]$	*Đ*	$M_{sD}10^{-3}\;[g/\mathrm{mol}]$
1 *	58	54	1.07	**43**
2 *	43	39	1.10	**35**
3 *	73	65	1.12	**32**
4 *	33	32	1.03	**25**
5	50	38	1.32	**20**
6	20	16	1.25	**17**
7 *	19	18	1.06	**16**
8	20	16	1.25	**10**
9	9.9	7.1	1.39	**10**
10 *	9.5	8.7	1.09	**9.5**
11 ^U^	8.5	7.7	1.10	**7.5**
12	13	12	1.08	**6.8**
13 ^S^	11	8.1	1.36	**5.4**
14 *	5.1	4.8	1.06	**4.2**
15	3.6	2.8	1.29	**2.3**
16	3.3	2.6	1.27	**1.6**
17	1.8	1.5	1.20	**1.2**

* The samples are obtained by acetylation of corresponding PEI [47], ^U^—Ultroxa, ^S^—Sigma-Aldrich.

**Table 2:** The table contains the results of viscous PMeOx solution study in PBS at 37 °C, interpreted within the framework of Huggins (${\left[\eta \right]}_{H}$, $k^{\prime }$ ) and Kraemer (${\left[\eta \right]}_{K}$, $k^{\prime \prime }$ ) procedures, together with the averaged values of intrinsic viscosity, $\left[\eta \right]$.

PMeOx Sample	${\left[\eta \right]}_{H}\;[{\mathrm{cm}}^{3}/g]$	$k^{\prime }$	${\left[\eta \right]}_{K}\;[{\mathrm{cm}}^{3}/g]$	$-k^{\prime \prime }$	$\langle \left[\eta \right]\rangle \;[{\mathrm{cm}}^{3}/g]$
1 *	60.7 ± 0.3	0.36	60.4 ± 0.1	0.14	60.5
2 *	55 ± 2	0.38	55.5 ± 0.9	0.14	55
3 *	52.4 ± 0.9	0.49	53.4 ± 0.4	0.10	52.9
4 *	41.1 ± 1.3	0.38	41.1 ± 0.7	0.14	41.1
5	27.3 ± 0.6	0.51	27.9 ± 0.1	0.10	27.6
6	23.5 ± 0.1	0.47	23.8 ± 0.1	0.11	23.7
7 *	30.1 ± 0.2	0.42	30.3 ± 0.1	0.12	30.2
8	19.1 ± 0.1	0.53	19.5 ± 0.1	0.09	19.3
9	16.6 ± 0.6	0.34	16.4 ± 0.4	0.15	16.5
10 *	18.7 ± 0.1	0.44	18.8 ± 0.1	0.10	18.8
11 ^U^	17.1 ± 0.1	0.56	17.4 ± 0.1	0.07	17.2
12	14.5 ± 0.1	0.58	14.7 ± 0.1	0.05	14.6
13 ^S^	11.5 ± 0.1	0.53	11.6 ± 0.1	0.07	11.6
14 *	-	-	-	-	11.9 ^2^
15	5.6 ± 0.1	0.62 ^1^	5.6 ± 0.1	0.03 ^1^	5.6
16	5.1 ± 0.1	0.69 ^1^	5.2 ± 0.1	0 ^1^	5.2
17	3.3 ± 0.1	1.3 ^1^	3.4 ± 0.1	-0.4 ^1^	3.4

^1^ The data were not included in averaging; ^2^ in the first approximation, the value was estimated by one concentration using the Solomon-Cuita equation [60].

**Table 3:** Hydrodynamic parameters ($\left[\eta \right],\;\langle D_{0}\rangle ,\;s_{0}$), hydrodynamic radiuses $R_{h}$, absolute molar masses $M_{\mathrm{sD}}$ and hydrodynamic invariants $A_{0}$ of PMeOx samples in PBS at 37 °C, together with the number of Kuhn segments N=L/A, where L —the contour length of a polymer chain and A —the Kuhn segment length (or equilibrium rigidity).

PMeOx Sample	[*η*] [cm^3^/g]	<*D*_0_>10^7^ [cm^2^/s] ^1^	*R*_h_ [nm]	*s*_0_10^13^ [s]	*M*_sD_10^−3^ [g/mol]	*A*_0_10^10^	*L*/*A*
1 *	60.5	5.5	5.8	1.81	43	3.7	110
2 *	55	6	5.3	1.61	35	3.7	90
3 *	52.9	6.8	4.7	1.66	32	4.0	80
4 *	41.1	7.2	4.5	1.39	25	3.6	65
5	27.6	7.7	4.2	1.19	20	3.1	50
6	23.7	8.9	3.6	1.16	17	3.2	40
7 *	30.2	9.3	3.4	1.1	16	3.6	42
8	19.3	10.8	3.0	0.85	10	3.1	30
9	16.5	11.5	2.8	0.87	10	3.1	30
10 *	18.8	12.4	2.6	0.89	9.5	3.4	23
11 ^U^	17.2	14.1	2.3	0.8	7.5	3.5	20
12	14.6	14.4	2.2	0.74	6.8	3.3	18
13 ^S^	11.6	18.4	1.7	0.75	5.4	3.6	14
14 *	11.9	18.5	1.7	0.59	4.2	3.4	11
15	5.6	31	1.0	0.53	2.3	3.6	6
16	5.2	39	0.8	0.48	1.6	3.9	4
17	3.4	41	0.8	0.37	1.2	3.2	3

^1^ The data are averaged based on obtained diffusion coefficients from DLS and AUC experiments.

**Table 4:** Parameters of scaling KMHS relationships for PMeOx in PBS at 37 °C and H_2_O at 20 °C.

$P_{i}-P_{j}$	$b_{ij}$	$K_{ij}$	$r_{ij}{\;}^{1}$
T=37 °C			
$\left[\eta \right],{\;\mathrm{cm}}^{3}/g\;-M_{\mathrm{sD}}$	0.77 ± 0.04	0.015 ± 0.004	0.9908
$s_{0}10^{13},\;s\;-M_{\mathrm{sD}}$	0.42 ± 0.02	0.019 ± 0.003	0.9911
$D_{0}10^{7},{\mathrm{cm}}^{2}/s\;-M_{\mathrm{sD}}$	−0.58 ± 0.02	2600 ± 400	−0.9949
T=20 °C			
$\left[\eta \right],{\;\mathrm{cm}}^{3}/g\;-M_{\mathrm{sD}}$	0.77 ± 0.03	0.017 ± 0.005	0.9882

^1^ The linear correlation coefficients of corresponding double logarithmic dependences (Figure 4a).

**Table 5:** The values of equilibrium rigidity A and effective hydrodynamic diameter d, calculated for PMeOx homologous series in PBS at 37 °C with different theoretical models.

Theory by	$A_{\eta }\;[\mathrm{nm}]$	$A_{D}\;[\mathrm{nm}]$	$d_{\eta }\;[\mathrm{nm}]$	$d_{D}\;[\mathrm{nm}]$
Yamakawa–Fujii	2.7	2.7	0.3	0.4
Fixman–Stockmayer	1.0 ± 0.1			
Cowie–Bywater		1.6 ± 0.1		
Gray–Bloomfield–Hearst	1.7 ± 0.1	1.7 ± 0.2	0.2 ± 0.1	0.4 ± 0.2

## Data Availability

The data on the synthesis and structural characterization of all compounds are stored at the Ghent University and St. Petersburg State University.

## Supplementary Materials

The following supporting information can be downloaded at: https://www.mdpi.com/article/10.3390/polym15030623/s1, Tables S1 and S2: Summarized data of PMeOx intrinsic viscosity in H_2_O at 20, 37, 60 °C and PBS at 37 °C. Table S3: Hydrodynamic invariant values obtained with DLS determined data ($D_{0}$) and AUC estimations ($D_{0sf}$); Table S4: The values of equilibrium rigidity A and effective hydrodynamic diameter d calculated for PMeOx homologous series in H_2_O at 20 °C with different theoretical models; Figure S1: Viscometry data PMeOx-5 in H_2_O at 20, 37, 60 °C; Figures S2 and S3: AUC and DLS data; Figures S4–S6: Yamakawa–Fujii, Fixman–Stockmayer, Gray–Bloomfield–Hearst model applications.
