# Supplementary material for: Conformational Parameters and Hydrodynamic Behavior of Poly(2-Methyl-2-Oxazoline) in a Broad Molar Mass Range
Source: Polymers (Basel). 2023 Jan 25;15(3):623. doi: 10.3390/polym15030623 (PMC9921015; doi:10.3390/polym15030623)
Supplement: Supplementary file 1 [file polymers-15-00623-s001.zip › polymers-2109565-supplementary.pdf]

Table S1. The table contains the results of viscous PMeOx solution study in H2O at 20, 37 and 60 °C, interpreted within the framework of Huggins ($[\eta]_H$, k') and Kraemer ($[\eta]_K$, k'') procedures, together with the averaged values of $[\eta]$ intrinsic viscosity.

**PMeOx sample**	$[\boldsymbol{\eta}]_{\mathbf{H}}$ [cm³/g]	$\boldsymbol{k'}$	$[\boldsymbol{\eta}]_{\mathbf{K}}$ [cm³/g]	$-\boldsymbol{k''}$	$< [\boldsymbol{\eta}] >$ [cm³/g]
			T = 20 °C		
1*	69.4±0.9	0.43	70.1±0.5	0.12	69.7
2*	60.9±1.3	0.35	60.8±0.9	0.15	60.9
3*	57.0±0.6	0.39	57.1±0.3	0.13	57.0
5	30.4±0.8	0.41	30.6±0.5	0.13	30.5
6	24.8±0.3	0.49	25.2±0.1	0.10	25.0
8	20.7±0.3	0.50	21.1±0.1	0.10	20.9
9	19.0±0.8	0.16	18.5±0.6	0.23	18.7
11^{U}	17.8±0.2	0.57	18.2±0.1	0.07	18.0
12	15.7±0.2	0.54	16.0±0.1	0.08	15.9
15 [1]	5.7±0.1	0.73	5.7±0.1	-0.08	5.7
16 [1]	5.2±0.1	0.90	5.3±0.1	-0.22	5.3
			T = 37 °C		
3*	55.7±1.3	0.33	55.3±0.7	0.15	55.5
5	28.5±0.9	0.38	28.6±0.6	0.14	28.5
8	19.5±0.2	0.47	19.8±0.1	0.11	19.6
12	14.9±0.2	0.50	15.1±0.1	0.09	15.0
			T = 60 °C		
3*	55±6	0.22	53±3	0.18	54
5	24.9±0.4	0.42	25.1±0.3	0.12	25.0
8	17.0±0.2	0.54	17.3±0.1	0.08	17.2
12	13.3±0.2	0.50	13.5±0.1	0.09	13.4

[1] The data was obtained in PBS at 20 °C.

Table S2. The table contains data on comparison of averaged Huggins and Kraemer intrinsic viscosities in H_2O at 20, 37, 60 °C and PBS at 37 °C.

PMeOx sample	$< [\eta]_{w20} >$, cm^3g^{-1}	$< [\eta]_{w37} >$, cm^3g^{-1}	$< [\eta]_{PBS37} >$, cm^3g^{-1}	$< [\eta]_{w60} >$, cm^3g^{-1}
	H_2O-20 °C	H_2O-37 °C	PBS-37 °C	H_2O-60 °C
3*	57.0±0.5	**55.5**±1.0	**52.9**±0.7	54±5
5	30.5±0.7	**28.5**±0.8	**27.6**±0.4	25.0±0.4
8	20.9±0.2	**19.6**±0.2	**19.3**±0.2	17.2±0.2
12	15.9±0.2	**15.0**±0.2	**14.6**±0.1	13.4±0.2

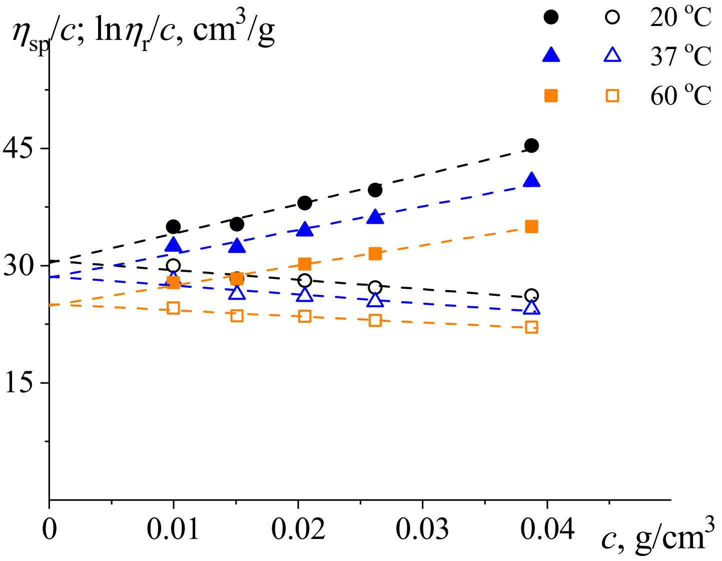

Figure S1. The normalized specific viscosity η_{sp}/c (filled symbols) and natural logarithm of relative viscosity $\ln\eta_r/c$ (open symbols) vs. concentration c obtained for PMeOx-5 in H_2O at 20, 37 and 60 °C.

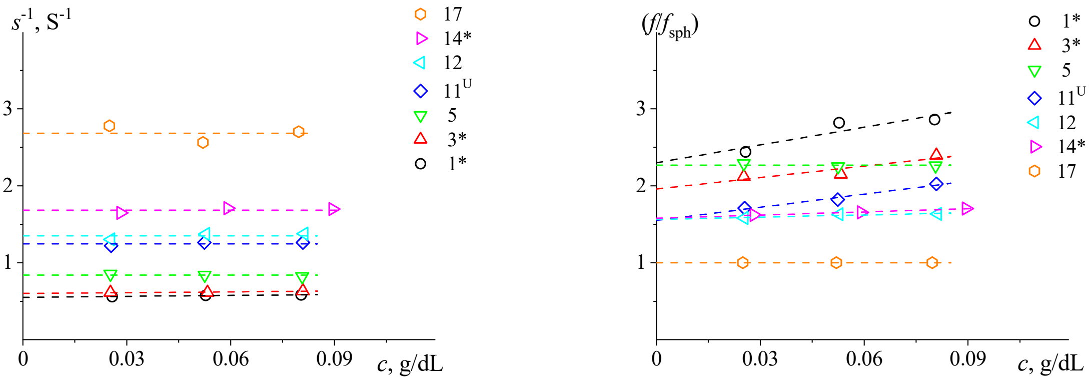

(**a**) (**b**)

Figure S2. The concentration dependence of reciprocal sedimentation **(a)** coefficients and frictional ratio **(b)** values resolved for PMeOx samples in PBS at 37 °C. The numbers next to the data points correspond to the sample numbering.

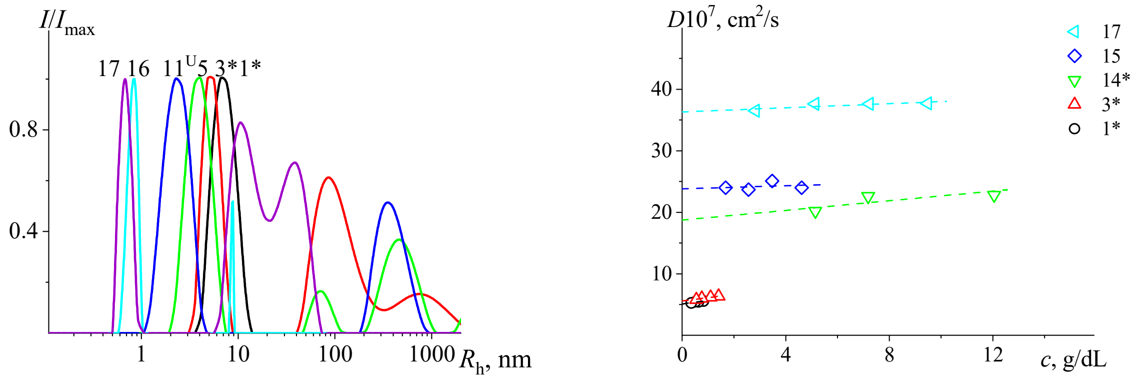

(**a**) (**b**)

Figure S3. (a): The normalized distributions of intensity-component concentration I/I_{max} over hydrodynamic radii R_h and **(b)**: the concentration dependences of diffusion coefficients obtained for PMeOx in PBS at 37 °C. The numbers next to the data points correspond to the sample numbering.

Table S3. Hydrodynamic invariant values obtained with DLS determined data (D_0) and AUC estimations (D_{0sf}).

**PMeOx sample**	$D_0 10^7$, cm²/s	$A_0 10^{10}$	$(f/f_{sph})_0$	$D_{0sf} 10^7$, cm²/s	$A_{0sf} 10^{10}$
1*	5.1	3.55	2.3	6.0	3.94
2*	5.2	3.33	2.2	6.8	3.99
3*	5.6	3.52	1.96	7.9	4.42
4*	6.0	3.17	2.0	8.4	3.99
5	7.9	3.17	2.27	7.5	3.08
6	7.8	2.98	1.9	9.9	3.49
7*	8.5	3.36	1.91	10.1	3.77
8	9.9	2.93	1.9	11.6	3.26
9	10.4	2.9	1.78	12.6	3.3
10*	10.7	3.11	1.65	14.0	3.72
11U	12	3.14	1.55	16.2	3.85
12	12	2.89	1.56	16.7	3.61
13S	13.1	2.86	1.23	23.7	4.25
14*	18.7	3.38	1.58	18.4	3.35
15	23.8	2.98	1	38.4	4.1
16	37	3.75	1	40.4	3.98
17	36.3	2.96	1	46.0	3.47

Table S4. The values of equilibrium rigidity A and effective hydrodynamic diameter d calculated for PMeOx homologous series in H_2O at 20 °C with different theoretical models.

Theory by	A_η, [nm]	A_D, [nm]	d_η, [nm]	d_D, [nm]
Fixman-Stockmayer	1.0 ± 0.1			
Cowie-Bywater		1.9 ± 0.2		
Gray-Bloomfield-Hearst [1]	1.7 ± 0.2	1.6 ± 0.2	0.3 ± 0.1	0.4 ± 0.2

[1] The thermodynamic solution parameter ε = (0.10 ± 0.02).

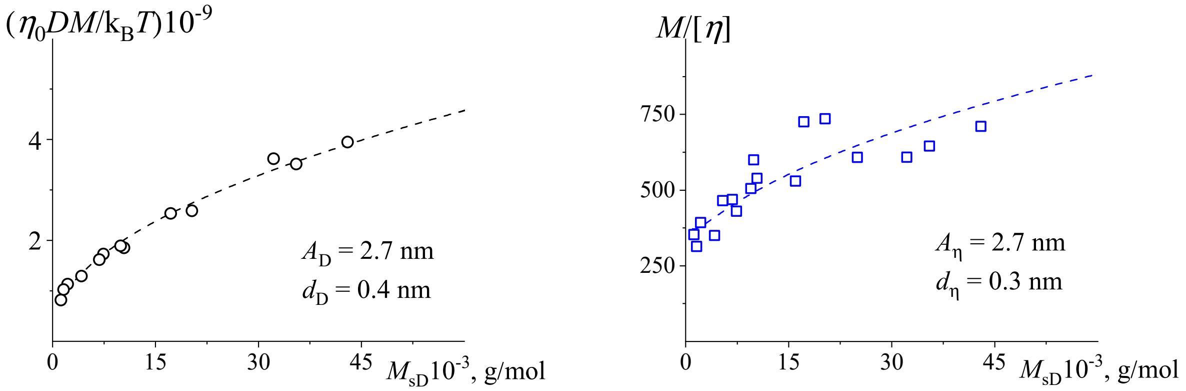

(**a**) (**b**)

Figure S4. The Yamakawa-Fujii theory application to obtained translation friction (**a**) and rotation friction (**b**) data for PMeOx in PBS at 37 °C.

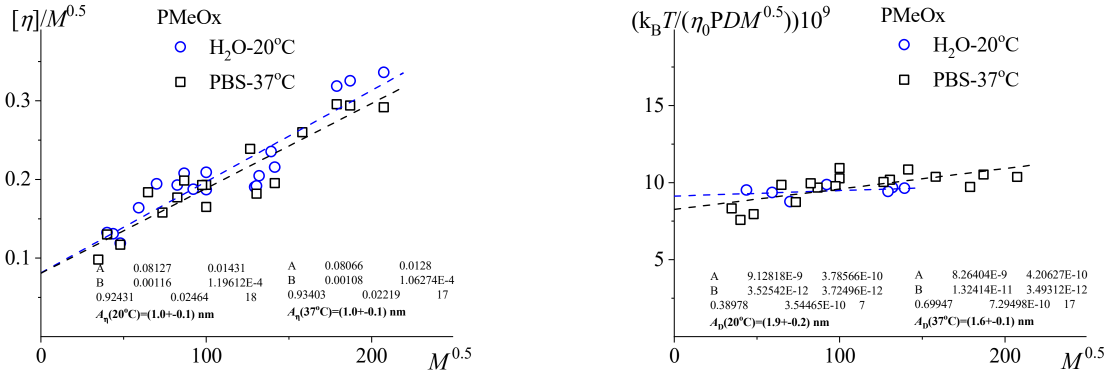

(**a**) (**b**)

Figure S5. The Fixman-Stockmayer (**a**) and Cowie-Bywater (**b**) extrapolation procedures for PMeOx in PBS at 37 °C (black squares) and H_2O at 20 °C (blue circles) based on combined analysis of newly obtained and previously reported data.

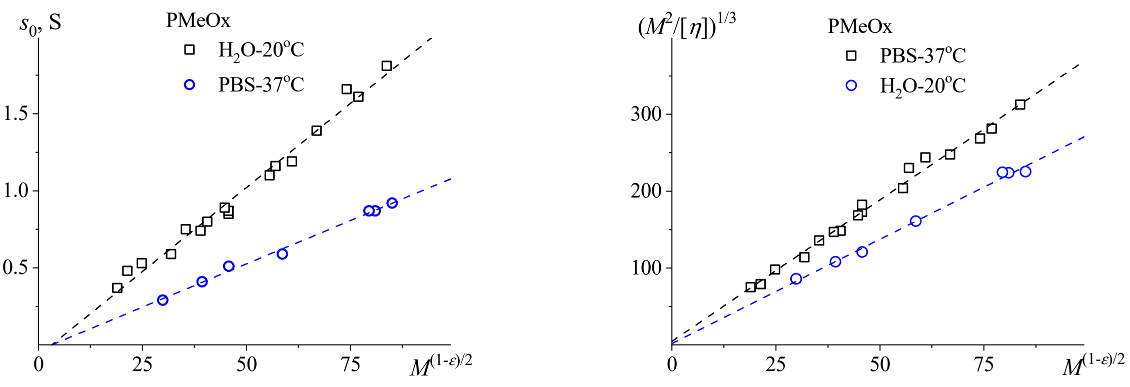

(**a**) (**b**)

Figure S6. The Gray-Bloomfield-Hearst theory application to obtained translation friction (**a**) and rotation friction (**b**) for PMeOx in PBS at 37 °C (black squares) and H_2O at 20 °C (blue circles) based on only the data reported earlier due to insufficient data for exact determination of thermodynamic solution parameter ε for performing the combined analysis at 20 °C.
